# ENVE: a novel computational framework characterizes copy-number mutational landscapes in colorectal cancers from African American patients

**DOI:** 10.1186/s13073-015-0192-9

**Published:** 2015-07-20

**Authors:** Vinay Varadan, Salendra Singh, Arman Nosrati, Lakshmeswari Ravi, James Lutterbaugh, Jill S. Barnholtz-Sloan, Sanford D. Markowitz, Joseph E. Willis, Kishore Guda

**Affiliations:** Division of General Medical Sciences-Oncology, Case Western Reserve University, Cleveland, OH 44106 USA; Case Comprehensive Cancer Center, Case Western Reserve University, Cleveland, OH 44106 USA; Division of Hematology and Oncology, Case Western Reserve University, Cleveland, OH 44106 USA; Department of Medicine, Case Western Reserve University, Cleveland, OH 44106 USA; Case Medical Center, Case Western Reserve University, Cleveland, OH 44106 USA; Department of Pathology, Case Western Reserve University, Cleveland, OH 44106 USA; Case Western Reserve University, 2103 Cornell Road, Wolstein Research Building, Cleveland, OH 44106 USA

## Abstract

Reliable detection of somatic copy-number alterations (sCNAs) in tumors using whole-exome sequencing (WES) remains challenging owing to technical (inherent noise) and sample-associated variability in WES data. We present a novel computational framework, ENVE, which models inherent noise in any WES dataset, enabling robust detection of sCNAs across WES platforms. ENVE achieved high concordance with orthogonal sCNA assessments across two colorectal cancer (CRC) WES datasets, and consistently outperformed a best-in-class algorithm, Control-FREEC. We subsequently used ENVE to characterize global sCNA landscapes in African American CRCs, identifying genomic aberrations potentially associated with CRC pathogenesis in this population. ENVE is downloadable at https://github.com/ENVE-Tools/ENVE.

**Electronic supplementary material:**

The online version of this article (doi:10.1186/s13073-015-0192-9) contains supplementary material, which is available to authorized users.

## Background

Human cancer is caused in part by structural changes resulting in DNA copy-number alterations at distinct locations in the tumor genome. Identification of such somatic copy-number alterations (sCNA) in tumor tissues has contributed significantly to our understanding of the pathogenesis and to the expansion of therapeutic avenues across multiple cancers [1–4]. Traditionally, sCNAs have been detected using cytogenetic techniques such as fluorescent in situ hybridization, array comparative genomic hybridization [5], and representational oligonucleotide microarrays [6] as well as single nucleotide polymorphism (SNP) arrays [7]. However, each of the above techniques has limitations with regard to the number, resolution, and platform-specific assessability of regions that can be interrogated in the genome.

More recently, massively parallel sequencing technologies have provided the unique opportunity to comprehensively characterize genome-scale DNA alterations in tumor tissues. In particular, whole-exome sequencing (WES) offers a cost-effective way of interrogating mutation and copy-number profiles within protein-coding regions in the tumor genome. This has resulted in the increasing use of WES in both the research [8, 9] and clinical settings [10, 11]. However, variability in tumor content among clinical samples in addition to the random technical variability in DNA library enrichment steps during WES can potentially introduce systematic biases across the genome, thus making sCNA detection relatively challenging. Although quite a few algorithmic approaches have been developed to address these issues [12–18], a recent comprehensive review [19] of these published methodologies, primarily using simulated data, showed substantial variability in sensitivity and specificity across algorithms, with algorithm-specific parameter choice a key confounder of algorithm performance. This poses a significant challenge in reliably detecting sCNAs in WES data because choosing the right parameter for a given application is non-trivial. There is therefore a pressing need to develop relatively parameter-free and robust methodologies for detecting these sCNAs across diverse tumor types and sequencing platforms.

Here we present a novel computational methodology, ENVE (**E**xtreme Value Distribution Based Somatic Copy-**N**umber **V**ariation **E**stimation), which robustly detects tumor-specific copy-number alterations in massively parallel DNA sequencing data without the need for complex parameter choices or user intervention. We demonstrate the robustness of ENVE’s performance in two independent matched tumor/normal WES datasets (total N = 107), derived from Caucasian and African American (AA) colorectal cancers (CRC), by comparing ENVE-based sCNA calls in WES data against SNP arrays and quantitative real-time PCR (qPCR)-based sCNA assessments performed on the same sample sets. We further show ENVE as significantly and consistently outperforming the best-in-class sCNA detection algorithm, Control-FREEC [12, 19], in these analyses. We additionally demonstrate the reproducibility of ENVE’s key noise-modeling feature using an independent WES dataset derived from 54 normal diploid samples. Finally, using the ENVE framework, we characterize, for the first time, global sCNA landscapes in colon cancers arising in AA patients, identifying genomic aberrations potentially associated with colon carcinogenesis in this population.

## Methods

### AA CRC samples

The AA CRC sample set included a total of 30 fresh-frozen, predominantly late-stage microsatellite stable (MSS) CRCs and matched normal samples from AA patients (Additional file 1: Table S1). The colon cancer diagnosis of all tumor samples was reviewed and confirmed by an anatomic pathologist (JW). Genomic DNA from the tumor samples was extracted as previously described [20]. DNA from all patients’ tumors was confirmed as being MSS by evaluation of microsatellite alleles in tumor and matched normal DNA at microsatellite markers BAT26, BAT40, D2S123, D5S346, and D17S250 [21]. All samples used in this study were accrued under the tumor sample accrual protocol entitled, “CWRU 7296: Colon Epithelial Tissue Bank,” which was approved by the University Hospitals Case Medical Center Institutional Review Board for Human Investigation with the assigned UH IRB number 03-94-105. Under this protocol, tissue was obtained through written informed consent from patients for research use. All aspects of this study were conducted in accordance with these approved guidelines.

### Whole-exome capture, deep sequencing, and alignment of AA CRC samples

Target capture, library preparation, and deep sequencing of the 30 normal/tumor paired frozen DNA samples were performed by the Oklahoma Medical Research Foundation Next Generation DNA Sequencing Core Facility (Oklahoma City, OK, USA). Target sequence enrichments were performed using the Illumina TruSeq Exome Enrichment kit as per the manufacturer’s protocols (Illumina Inc., San Diego, CA, USA). Briefly, sample DNA was quantified using a PicoGreen fluorometric assay, and 3 μg of genomic DNA was randomly sheared to an average size of 300 bp using a Covaris S2 sonicator (Covaris Inc., Woburn, MA, USA). Sonicated DNA was then end-repaired, A-tailed, and ligated with indexed paired-end Illumina adapters. Target capture was performed on DNA pooled from six indexed samples, following which the captured library was PCR amplified for ten cycles to enrich for target genomic regions. The captured libraries were precisely quantified using a qPCR-based Kapa Biosystems Library Quantification Kit (Kapa Biosystems Inc., Woburn, MA, USA) on a Roche LightCycler 480 (Roche Applied Science, Indianapolis, IN, USA). Deep sequencing of the capture enriched DNA pools was performed on an Illumina HiSeq 2000 instrument to generate 100-bp paired-end reads, and to achieve an average read depth of ~70× per tumor sample and ~50× per matched normal sample. A Burrows–Wheeler Aligner version 0.6.1-r104 [22] algorithm [23] was used to align individual 100-bp reads from the raw FASTQ files to the human reference genome (build hg19). Following the conversion of aligned reads in to Binary Sequence Alignment/Map (BAM) format and subsequent removal of duplicated reads, coverage metrics of target bases were calculated using the Picard tools version 1.41 [24]. On average, Picard metrics showed ~69 % of the target bases covered at 20× read depth for the normal samples and ~86 % of the target bases covered at 20× read depth for the tumors.

### The Cancer Genome Atlas CRC whole-exome dataset

We identified a total of 77 MSS colon adenocarcinoma and matched normal WES samples from The Cancer Genome Atlas (TCGA) colon cancer repository on the Cancer Genomics Hub [25], for which Affymetrix SNP6 array-based copy-number profiles were available on TCGA Data Portal (Additional file 1: Table S1). BAM files for the 77 tumor/normal pairs were downloaded from Cancer Genomics Hub using the GeneTorrent client. Subsequent to removal of duplicated reads, coverage metrics of target bases were calculated using the Picard tools. On average, Picard metrics showed ~86 % of the target bases covered at 20× read depth for both the normal and tumor samples.

### SNP array-based copy-number analysis

We evaluated 12 of the 30 AA tumor/normal paired samples (Additional file 1: Table S1) for genome-wide somatic copy-number alterations using HumanOmni2.5-8 BeadChips containing 2,379,855 markers (Illumina). Briefly, 200 ng of normal and tumor DNA were hybridized on to the BeadChips and array images were scanned using the HiScan System (Illumina). The array data were subsequently processed using GenomeStudio software to generate the B-allele frequency and log-ratio values across all the markers per individual chromosome (Illumina). Quality control analysis of the SNP array data revealed an average of 98 % (range 91–99 %) call rate for the samples. The B-allele frequency and log-ratio values of the samples were next imported into Partek Genomics Suite software (Partek Inc., St. Louis, MO, USA) to identify regions showing significant copy-number alterations in the tumors. This analysis was performed as per the manufacturer’s instructions using default settings on the genomic segmentation algorithm, which included a minimum marker-distance of 50, *P*-value threshold of 0.001, and signal to noise ratio of 0.3.

Affymetrix SNP Array 6.0–based copy-number profiles were obtained from TCGA portal [26] for the 77 TCGA CRC WES samples (Additional file 1: Table S1). TCGA Level 3 copy-number data provide segmented copy-number calls, after elimination of potential germline copy-number variation (CNV) in each sample, using the Broad Institute’s Copy Number Inference pipeline for Affymetrix SNP Array 6.0 arrays. For each tumor sample, genome-wide segmented copy-number calls were obtained at different Segment-Mean cutoff values ranging from ≥0.1 to ≥0.5 (indicating somatic amplifications) and from ≤−0.1 to ≤−0.5 (indicating somatic deletions). In all cases, segmented copy-number calls inferred from at least 10 SNP array probes were included in the analyses as previously suggested [2, 27].

### Copy-number analysis using pooled normals

For the AA CRC WES dataset, we first computationally pooled reads from the 30 AA normal samples (Additional file 1: Table S1). Next, we sub-sampled this pooled normal data to generate 12 independent reference normals containing a similar number of total mapped reads observed for each of the 12 AA tumor samples for which SNP array data were available. We then performed ENVE Modules 2a-c on this simulated data to identify significant (ENVE *P* ≤ 0.05) sCNAs in the 12 AA tumor samples. Similarly, for the SNP array data, a computationally pooled reference normal was derived from the 12 AA normal samples using the Partek Genomics Suite software (Partek) followed by SNP array-based sCNA detection in the 12 tumors.

### qPCR-based estimation of somatic copy-number alterations

Recurrent somatic copy-number alterations in candidate regions identified by ENVE in the WES dataset were validated using a qBiomarker qPCR copy-number array as per the manufacturer’s instructions (Qiagen Inc., Valencia, CA, USA). Briefly, 500–700 ng of genomic DNA from six matched tumor/normal AA CRC cases used for WES, and DNA from an additional six AA normal colon samples (Additional file 1: Table S1) was used for qPCR validation of a custom 11-gene panel, with each gene mapping to a distinct genomic locus. Of note, each of the 11 genes on the custom panel selected for qPCR analysis showed significant copy-number alteration, as detected by ENVE in WES data, in at least one of the six AA CRC cases. Pre-designed qPCR primers for the 11 candidate genes and a multi-copy reference (MRef) control were plated in quadruplicate on a 96-well plate, enabling the analysis of two samples per plate (Qiagen). qPCR was carried out using the CFX96 Real-Time PCR equipment (BioRad, Hercules, CA, USA) in a total volume of 25 μl containing the SYBR Green Assay Master Mix (Qiagen) for 10 min at 95 °C, followed by 40 cycles of 95 °C for 15 s and 60 °C for 1 min. Cq values obtained from each of the reaction wells were uploaded to an online data analysis tool [28] for subsequent significance estimation of tumor-specific CNAs in the 11 target genes using the calibrator genome methodology, where the 12 AA normal samples served as diploid genome controls. Tumor-specific CNAs with *P* ≤ 0.05 were considered significant.

### Control-FREEC–based copy-number analysis for AA and TCGA CRC datasets

Somatic copy-number analysis on the AA CRC and TCGA CRC WES datasets was performed using the developer’s recommended parameters for processing WES data from matched tumor/normal samples [12]. The window size was set to 500 bp with a step of 250 bp for all of the analyses. GC content normalization was enabled for all of the analyses, along with the noisyData option set as TRUE in order to avoid false-positive predictions due to non-uniform capture in exome sequencing data. For the primary analyses, Control-FREEC (version 6.7) was run in the default mode without enabling correction for contamination by normal cells. However, contamination adjustment was subsequently enabled to evaluate whether automatic inference of tumor content in the tissue samples improved the performance of Control-FREEC.

### Recurrent sCNA identification using GISTIC

The ENVE output file containing ENVE *P-*values assigned to each candidate copy-number–altered segment in the 30 AA CRC and stage-matched 30 TCGA Caucasian CRC cases was analyzed using the GISTIC tool (version 2.0.21) [27]. The markers file for GISTIC was derived as the union of the start coordinates of all possible 100-bp segments within the exonic regions defined in the region-of-interest file for the Illumina TruSeq Exome platform. Copy-number–altered segments that were not considered significant (ENVE *P* > 0.05) were assigned a LogRatio value of 0, thus making them copy-neutral for GISTIC analysis. GISTIC broad-level analysis was performed with a size threshold of 98 % of a chromosome arm to differentiate between arm-level and focal events. sCNA regions and arm-level events with q ≤ 0.25 were considered significant. The significance of focal sCNA events was determined using residual q-values, which were estimated by removing amplifications or deletions that overlapped other, more significant sCNAs in the same chromosome. Focal sCNAs with residual q ≤ 0.05 were considered significant. The frequencies of the resulting significant recurrent sCNAs were plotted using ggplot2 (R package version 0.9.3.1).

### WES data accessibility for AA CRC and TCGA cohorts

As mentioned above, the 77 MSS colon adenocarcinoma and matched normal WES samples from TCGA colon cancer cohort are publicly available in the repository on the Cancer Genomics Hub [25]. The AA CRC WES dataset (N = 30) was generated in-house, and all appropriate processed files relevant to this study can be accessed at the ENVE Tool website [29].

## Results

We describe the key computational steps in the ENVE methodology and evaluate its performance using two matched tumor/normal WES datasets, an in-house WES dataset of predominantly late-stage, MSS AA CRCs (N = 30) [20], and a Caucasian MSS CRC WES dataset obtained from TCGA (TCGA CRC, N = 77) (See “Methods” and Additional file 1: Table S1). A subset of these TCGA cases consisting of predominantly late-stage cancers (N = 30) was used to further assess differences in sCNAs in CRCs arising in AA versus Caucasian ethnicities.

### ENVE methodology overview

**Fig. 1:**
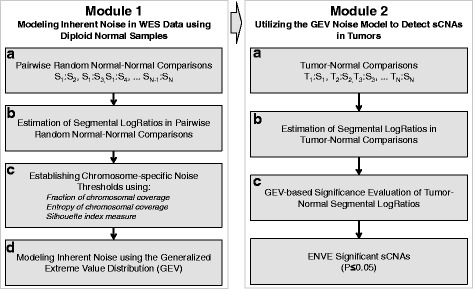
Overview of the ENVE workflow to detect somatic copy-number alterations. The ENVE framework consists of two major modules: the first involves modeling of inherent noise in WES data using normal diploid samples (Module 1 on the left); the second module utilizes the expected variability as captured by the learned model parameters to detect sCNAs in tumors (Module 2 on the right)

The ENVE methodology consists of two major modules: Module 1 uses non-tumor normal diploid samples to capture and model inherent noise in WES data likely arising from technical variability in the DNA capture, hybridization, and/or amplification steps, in addition to variability in sequencing platforms. This is followed by Module 2. which utilizes the learned model parameters to reliably detect somatic copy-number alterations in tumors (Fig. 1).

#### Module 1

Module 1 of the ENVE methodology consists of four steps as follows:

#### Module 1a: Pairwise random normal-normal comparisons

In this module (Fig. 1), WES profiles of *N* normal samples {S_1_, S_2_, …, S_N_} are taken pairwise, resulting in (*N* !)/(2 * (*N* − 2) !) random normal–normal combinations {S_1_:S_2_, S_1_:S_3_, …, S_N-1_:S_N_}. We accordingly applied this module to the WES profiles of the 30 AA normal and 30 TCGA normal samples, resulting in a total of 435 random normal–normal comparisons for the AA and TCGA cohorts.

#### Module 1b: Estimation of segmental LogRatios in pairwise random normal-normal comparisons

In this module (Fig. 1), genome-wide segmental LogRatios for each of the (*N* !)/(2 * (*N* − 2) !) random normal–normal combinations are calculated using read depth comparison and circular binary segmentation [30]. For each sample pair being compared, each target region within the exome is divided into non-overlapping 100-bp windows. The ratio of average read depth within these 100-bp windows is estimated for each pair of samples being compared (*D*_*Si*_ and *D*_*Sj*_) after normalizing for the total number of uniquely mapped bases per sample (*TR*_*Si*_ and *TR*_*Sj*_) as follows:

1 $$ {W}_{RdRatio}=lo{g}_2\left(\frac{D_{Si}}{D_{Sj}}*\frac{T{R}_{Sj}}{T{R}_{Si}}\right) $$

The ratio of read depth per window (*W*_*RdRatio*_) is then corrected for GC content according to published methodology [14]. The resulting GC-corrected *W*_*RdRatio*_ data are segmented using circular binary segmentation, resulting in genomic segments and associated segmental LogRatios summarized from the GC-corrected *W*_*RdRatio*_ of all 100-bp windows within each segment. The resulting distribution of genome-wide segmental LogRatios in the normal–normal comparisons is adjusted to be distributed around zero by subtracting the mode of the distribution from each of the segmental LogRatios.

**Fig. 2:**
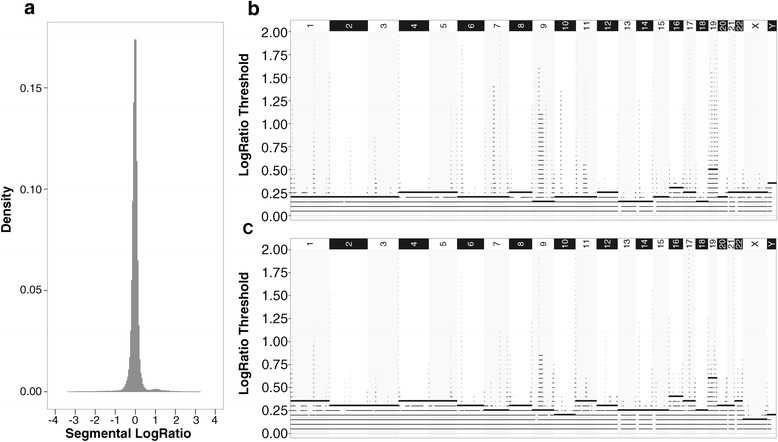
Modeling of inherent noise in the AA CRC WES dataset. **a** Distribution of exome-wide segmental LogRatios generated using 435 random normal–normal comparisons derived from 30 normal diploid WES samples. **b**, **c** Chromosomal coverage by copy-number altered segments at different LogRatio thresholds using the 435 normal–normal comparisons across all chromosomes for the positive (**b**) and negative (**c**) LogRatio deviations. *Bold horizontal lines* within each chromosome indicate the noise-threshold values

**Fig. 3:**
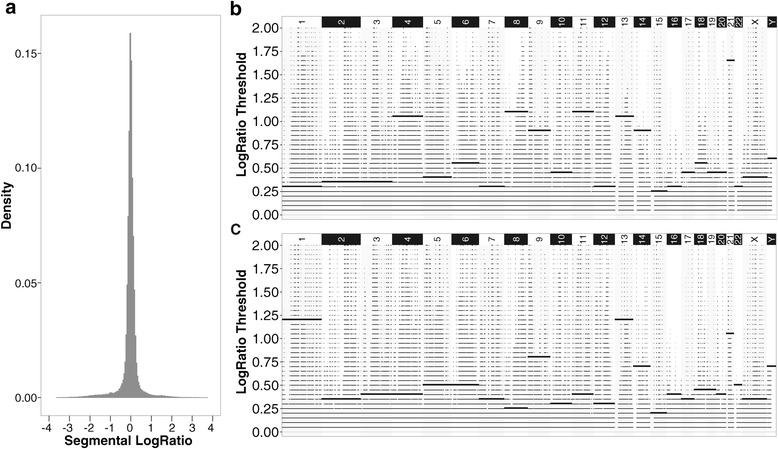
Modeling of inherent noise in the TCGA CRC WES dataset. **a** Distribution of exome-wide segmental LogRatios generated using 435 random normal–normal comparisons derived from 30 normal diploid WES samples. **b**, **c** Chromosomal coverage by copy-number altered segments at different LogRatio thresholds using the 435 normal–normal comparisons across all chromosomes for the positive (**b**) and negative (**c**) LogRatio deviations. *Bold horizontal lines* within each chromosome indicate the noise-threshold values

Accordingly, we applied ENVE Module 1b on the 435 random normal–normal comparisons derived from the AA and TCGA normal WES datasets. As expected for comparisons of normal diploid samples, the vast majority of the segmental LogRatios across the normal–normal comparisons were distributed around zero, with a minority of segments showing segmental LogRatios deviating significantly from zero (Figs. 2 and 3). Because these significant deviations could result from either inherent noise in WES data or focal germline CNV within the normal diploid samples being compared, identification of those segmental LogRatio deviations associated primarily with inherent noise is essential for subsequent noise modeling in WES data.

#### Module 1c: Establishing chromosome-specific noise thresholds

ENVE Module 1c (Fig. 1) is specifically designed to identify chromosome-specific segmental LogRatio deviations associated particularly with random inherent noise in WES data. Given that segmental LogRatio deviations in the normal–normal sample comparisons tend to be asymmetrically distributed around zero (for example, Figs. 2a and 3a), the positive and negative LogRatio deviations in the normal–normal comparisons are modeled separately. Accordingly, each chromosome is first divided into equal-sized non-overlapping 10-kb windows. The frequency with which each of the chromosomal windows is covered by copy-number altered segments at LogRatios ranging from 0 to 2 in increments of 0.05 is counted, both in the positive and negative directions. Because chromosomal coverage tends to be more complete and randomly distributed at lower Absolute LogRatio Thresholds (as expected with random inherent noise) for both positive and negative deviations, as opposed to the sparse and focal coverage observed at higher Absolute LogRatio Thresholds (as expected with germline CNVs), ENVE Module 1c employs a robust quantitative approach to differentiate between inherent noise and germline CNVs in the normal–normal comparisons (ENVE Module 1c, Fig. 1). Each chromosome is first divided into non-overlapping 10-kb windows. Subsequently, the frequency of segmental coverage within each chromosomal window is calculated using segments with absolute LogRatios at or above Absolute LogRatio Threshold (*R*_*T*_), with *R*_*T*_ varying from 0 to 1 (*R*_*Tmax*_) in steps of 0.05. Let *F*^*RT*^ be the vector containing the frequencies of segmental coverage $\left({f}_j^{R_T}\right)$ for all the windows in a chromosome at a particular *R*_*T*_. The *fraction of chromosomal coverage* at a particular *R*_*T*_ is therefore the ratio of non-zero entries in ${F}^{R_T}$ to the length of ${F}^{R_T}$ for a given chromosome. Next, the *entropy of chromosomal coverage*, for given a chromosome, at each *R*_*T*_ is given as:

2 $$ {E}_{R_T}\kern0.5em =\kern0.5em -\kern0.5em {\displaystyle \sum_{\forall j}}{f}_j^{R_T}*{ \log}_2\left({f}_j^{R_T}\right) $$

*R*_*TF*_ is defined as the *R*_*T*_ associated with the maximal drop in fraction of chromosomal coverage and *R*_*TE*_ is defined as the *R*_*T*_ associated with the first major loss in entropy of chromosomal coverage per chromosome. The maximum of *R*_*TF*_ and *R*_*TE*_ corresponds to the noise threshold (*R*_*NT*_), above which we expect to see focal alterations associated with germline CNVs, and below which we expect to see variations associated with random inherent noise. The *average silhouette index* [31] ascertains whether the pairwise distances between ${F}^{R_T}$ vectors across *R*_*NT*_ are substantially different from the pairwise distances between ${F}^{R_T}$ vectors within all *R*_*T*_ above or below *R*_*NT*_. Positive silhouette index values close to 1 suggest that the chromosome-specific noise thresholds (*R*_*NT*_*)* appropriately capture the variability associated with inherent noise.

Accordingly, we employed the above measures in ENVE Module 1c on the 435 AA and TCGA normal–normal comparisons, obtaining chromosome-specific noise thresholds (Figs. 2b,c and 3b,c; Additional file 2: Figures S1 and S2). These chromosome-specific noise thresholds exhibited high positive silhouette indices (≥0.8) in both the AA and TCGA normal–normal comparisons, indicating that the focal copy-number altered segments observed above the noise thresholds are qualitatively distinct from the random distribution of segments below the noise thresholds.

**Fig. 4:**
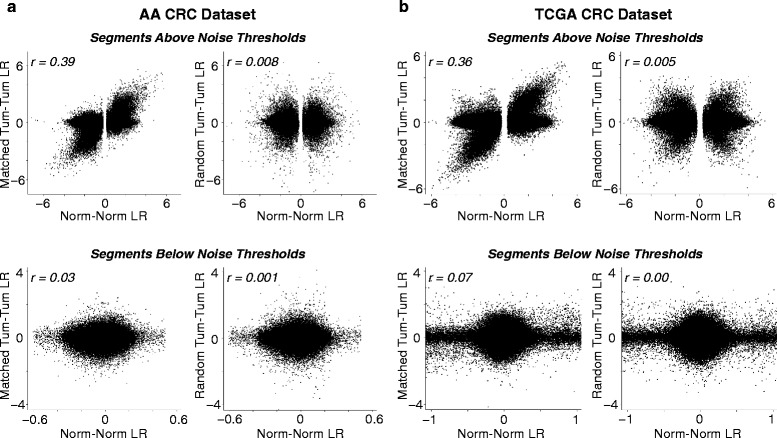
Ability of ENVE in distinguishing germline copy-number variation from random inherent noise. Segments falling above or below the estimated noise thresholds in normal–normal comparisons are evaluated against segments that overlap with the corresponding genomic regions from tumor–tumor comparisons in AA CRC (**a**) and TCGA CRC WES (**b**) datasets. Shown are the Pearson correlation coefficients comparing normal–normal segmental LogRatios with corresponding matched tumor–tumor or random tumor–tumor segmental LogRatios. Note the genomic segments in normal–normal comparisons falling above noise thresholds show higher correlation with segments in corresponding matched tumor–tumor comparisons than with segments in random tumor–tumor comparisons. By contrast, genomic segments in normal–normal comparisons that fall below noise thresholds show no correlation with either matched or random tumor–tumor comparisons

To further ascertain that these discrete focal alterations in genomic segments observed above the noise thresholds in the normal–normal data are indicative of germline CNVs prevalent among the normal samples, we repeated the analysis of ENVE Modules 1a-c (Fig. 1) by replacing the normal–normal comparisons with corresponding matched tumor–tumor comparisons for both the AA and TCGA CRC datasets, assuming that any likely germline CNV should also be detectable in the matched tumor samples. We next identified the genomic regions from the matched tumor–tumor comparisons overlapping the genomic segments falling above the noise thresholds in the normal–normal comparisons (Figs. 2b,c and 3b,c). Intriguingly, despite the potential confounding effects of somatic alterations as well as platform-associated noise in these tumor samples, we found that segments with LogRatios above the noise thresholds in the normal–normal comparisons exhibited significantly higher correlation with their matched tumor–tumor counterparts in both the AA CRC (Pearson’s *r* = 0.39; Fig. 4a) and TCGA CRC (Pearson’s *r* = 0.36; Fig. 4b) datasets, as compared to randomly selected tumor–tumor comparisons that showed virtually no correlation (Pearson’s *r* = 0.008 in AA CRC, Fig. 4a; Pearson’s *r* = 0.005 in TCGA CRC, Fig. 4b). However, segments with LogRatios below the noise thresholds in the normal–normal comparisons exhibited almost no correlation with either their matched tumor–tumor counterparts (Pearson’s *r* = 0.03 in AA CRC; Pearson’s *r* = 0.07 in TCGA CRC) or randomly selected tumor–tumor comparisons (Pearson’s *r* = 0.001 in AA CRC, Fig. 4a; Pearson’s *r* = 0.000 in TCGA CRC, Fig. 4b).

Taken together, these findings in two independent WES datasets strongly indicate that the focal copy-number altered segments falling above the noise thresholds are more likely to be associated with germline CNVs, whereas the randomly distributed segments falling below the noise thresholds are indicative of inherent noise in WES data. Additionally, we note that the focal genomic segments with high LogRatios above noise thresholds within each chromosome were repeatedly observed across multiple unique normal sample pair comparisons in both the AA and TCGA CRC datasets, providing further evidence of their being indicative of germline CNVs as opposed to random inherent noise. Importantly, the observed differences in chromosome-specific noise-threshold values in AA versus TCGA CRC datasets (Figs. 1 and 2) further highlight ENVE’s ability to model noise in a platform- and sample-agnostic manner.

#### Module 1d: Modeling inherent noise using the Generalized Extreme Value distribution

This module (Fig. 1) derives generalized extreme value (GEV) distribution-based models of the inherent noise associated with WES data. First, copy-number altered segments falling below the noise thresholds in the normal–normal comparisons (Figs. 2 and 3) are selected for noise modeling. Assuming *X*_*1*_, *X*_*2*_, … *X*_*n*_ to be the segmental LogRatios of selected copy-number altered segments within the normal–normal comparisons, the Fisher–Tippett theorem [32] states that the distribution of *M*_*n*_ = *max*{*X*_1_, *X*_2_, …, *X*_*n*_} converges to (as *n* → ∞) the GEV distribution:

3 $$ G(y)= exp\left(-{\left[1+\xi \left(\frac{y-\mu }{\sigma}\right)\right]}^{-1/\xi}\right) $$

where *ξ*, *μ*, and σ are the shape, the location, and scale parameters, respectively, that fully define the GEV. Because the only requirement for the GEV distribution is that the segmental LogRatios, *X*_*i*_, are independent and identically distributed random variables, the tails of whose distributions can have either an exponential or polynomial decay, we modeled the maxima of the segmental LogRatios using the GEV. Also, because the variability in segmental LogRatio estimates is likely to be chromosome-specific, reflecting variations in gene density and capture efficiency across regions, separate GEV model parameters are inferred for respective chromosomes. Furthermore, because somatic copy-number deletion events can only fall into two categories (heterozygous or homozygous deletions), as opposed to the copy-number amplifications, separate GEV parameters for positive and negative deviations are estimated, respectively, using the probability weighted moment method [32]. Accordingly, per chromosome, the maximum segmental LogRatio values associated with positive deviations within each of the *K* normal–normal comparisons, resulting in *K* maxima, are used to estimate the GEV parameters to evaluate somatic copy-number amplifications. Similarly, the minimum segmental LogRatio values associated with negative deviations within a chromosome for each of the *K* normal–normal comparisons, resulting in *K* minima, are used to estimate the GEV parameters to evaluate somatic copy-number deletions. The R package *fExtremes* (R package version 3010.81) is used to estimate the above GEV parameters.

We applied ENVE Module 1d on the AA and TCGA normal–normal comparisons to obtain chromosome-specific GEV parameters, thus effectively capturing and modeling chromosome-specific inherent noise associated with each of these WES datasets.

#### Module 2

We next applied ENVE Module 2 (Fig. 1) to call sCNAs in AA CRC and TCGA CRC WES samples.

#### Module 2a-b: Estimation of segmental LogRatios in tumor–normal comparisons

ENVE Module 2a-b performs read depth comparison and circular binary segmentation to identify all of the potentially copy-number altered segments along with their respective GC-corrected segmental LogRatios for each matched tumor/normal comparison similar to Module 1a-b. To account for potential aneuploidy/hyperploidy [33, 34] in the tumor samples, which could result in the segmental LogRatios of copy-neutral regions deviating from zero, the distribution of genome-wide segmental LogRatios in every tumor–normal comparison is adjusted by subtracting the mode of the distribution from each of the segmental LogRatios. Using these modules, we accordingly obtained segmental LogRatios for each of the AA and TCGA matched tumor/normal comparisons.

#### Module 2c: GEV-based significance evaluation of tumor–normal segmental LogRatios

The chromosome-specific GEV parameters for amplifications and deletions, as derived in Module 1d above, are used in Module 2c to evaluate the probability that an observed candidate amplification or deletion within a chromosome is due to inherent noise in WES data. This module employs the *pgev* function within *fExtremes* (R package version 3010.81). Segments that achieve a significant probability (*P* ≤ 0.05) are accordingly classified as being amplified or deleted in the respective tumor sample. Thus, by applying ENVE Modules 2a-c (Fig. 1), we identified chromosomal regions showing significant copy-number alterations (ENVE *P* ≤ 0.05) in each of the 30 AA CRC and 77 TCGA CRC samples (Additional file 1: Tables S2 and S3).

#### ENVE implementation

ENVE is implemented as a tool that is freely available along with the source codes for academic use at [29]. ENVE can accept BAM files and then performs the above outlined statistical analyses and outputs somatic copy-number alterations along with their segmental LogRatios and significance estimates for each tumor sample. The computational resources required to run ENVE using GC-corrected normalized read-counts are lightweight, wherein all of the normal–normal and tumor–normal analyses for a cohort of 77 tumor/normal samples could be performed on a desktop with a single processor and 16GB of memory in under 10 h.

### Evaluation of ENVE performance in detecting sCNAs in tumor samples

We next proceeded to systematically evaluate the performance of ENVE by assessing its sensitivity and specificity on individual tumor samples. Although there exists no gold-standard technique for use as a comparator in formal evaluation of sensitivity and specificity of sCNA calls in stromal-admixed clinical tumor samples, we nevertheless proceeded to evaluate ENVE’s performance by comparing against widely used SNP arrays.

Accordingly, we performed SNP array-based sCNA detection in both the AA CRC and TCGA CRC datasets. For the TCGA CRC dataset, we obtained SNP array-based sCNA calls for all of the 77 tumors included in our WES study from the TCGA portal (see “Methods”). Similarly, for the AA CRC dataset, we obtained SNP array sCNA calls in 12 of the 30 AA CRC samples used in our WES study (see “Methods”). As an additional key comparator, we evaluated another algorithm, Control-FREEC [12], which has been reported to outperform published WES-based sCNA detection algorithms in a comprehensive review [19]. We performed sCNA detection on the two WES datasets using Control-FREEC’s recommended parameters (see “Methods”), and subsequently compared the ENVE-based and Control-FREEC-based sCNA calls in each of the AA CRC and TCGA CRC samples, individually, with those detected by the SNP arrays. We anchored the comparison to only within gene-coding regions because SNP arrays also span substantial non-coding regions that are not interrogated by the WES platform. Because SNP arrays are also not a gold-standard technique for evaluating sensitivity and specificity, we instead assessed the concordance between the SNP arrays and the respective ENVE and Control-FREEC algorithms. Specifically, as shown in Additional file 2: Figure S3, the percent concordance between SNP arrays and ENVE/Control-FREEC was calculated as the ratio of the total length of all concordant exonic sCNA regions called by the WES algorithm to the total length of the exonic SNP array sCNA regions.

**Fig. 5:**
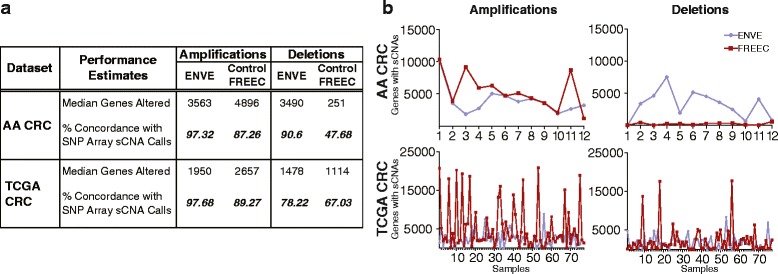
SNP array-based performance estimates of ENVE and Control-FREEC in detecting sCNAs in tumors. **a** Median number of genes with sCNAs, as detected by ENVE and Control-FREEC in the WES data, along with their median percent concordance with SNP array-based sCNA calls. **b** Number of genes with sCNAs (Y-axis) in each sample (X-axis), as detected by ENVE (blue) and Control-FREEC (red), in AA and TCGA CRC WES datasets. Significant sCNAs as detected by the Partek Suite were used for comparison in AA CRCs, while for TCGA CRCs SNP array segments with Segment-Mean cutoffs of ±0.5 were used for comparison

Figure 5a shows the median number of genes associated with sCNA regions in AA and TCGA CRC WES datasets, as detected by ENVE and Control-FREEC, along with their concordance with SNP array-based estimates. For regions with copy-number amplifications, ENVE achieved a higher concordance with SNP arrays than Control-FREEC both in the AA CRC (97.32 % vs. 87.26 %) and TCGA CRC (97.68 % vs. 89.27 %) datasets, despite Control-FREEC calling on average 30 % more amplification events than ENVE in both WES datasets (Fig. 5b). This strongly implies that ENVE has higher sensitivity and specificity in calling copy-number amplifications than Control-FREEC. Similarly, for regions with copy-number deletions, ENVE achieved a higher concordance rate with SNP arrays (Fig. 5a) than Control-FREEC both in the AA CRC (90.6 % vs. 47.68 %) and TCGA CRC datasets (78.22 % vs. 67.03 %), with Control-FREEC calling on average 16 % more deletion events than ENVE across samples in the TCGA CRC dataset (Fig. 5b). Noting the extremely poor performance of Control-FREEC in identifying deletion events, especially in the AA CRC WES dataset, we proceeded to evaluate whether its performance could be improved by enabling Control-FREEC to infer and adjust for potential stromal admixture and tumor content in the two WES datasets. In this mode, we found Control-FREEC called on average 55 % more copy-number altered events than ENVE, but did not match ENVE’s sensitivity in detecting sCNAs in three of the four comparisons across the AA and TCGA WES datasets (Additional file 2: Figure S4A,B). Moreover, ENVE consistently showed better performance than Control-FREEC when tested across different Segment-Mean cutoff values that were used for classifying sCNAs in the TCGA SNP array dataset (Additional file 2: Figure S5), or at low overall read depth simulated scenarios (Additional file 2: Figure S6). Taken together, these results strongly point to the potentially high sensitivity and specificity of the ENVE framework in detecting sCNAs in WES data.

**Fig. 6:**
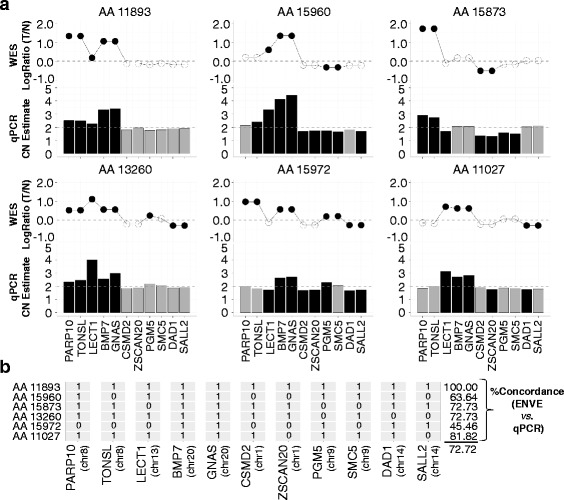
Concordance analysis of ENVE-based and qPCR-based sCNA estimates. **a** Matched tumor/normal (*T/N*) LogRatios derived from WES data (above) and corresponding qPCR-based copy-number (*CN*) estimates (below) for 11 genes in each of the six AA CRC cases. ENVE-significant LogRatios are indicated by *solid black circles*, with LogRatios above 0 indicating amplifications and LogRatios below 0 indicating deletions. Significant qPCR-based CN alterations are indicated by *solid black bars*, with values above 2 representing amplifications and values below 2 representing deletions. **b** Matrix showing concordance (*1*) and discordance (*0*) between ENVE and qPCR CN estimates for the 11 genes in each of the six AA CRC cases. For each case, copy-neutral genes by both ENVE and qPCR analyses, as well as genes showing significant CN alterations in the same direction by both platforms, were deemed concordant (from a). The resulting percent concordance between ENVE-based and qPCR-based CN estimates in each sample is shown on the right, with an overall concordance rate of 72.72 %

In addition to the above SNP array-based comparative analysis, we also evaluated ENVE’s performance by using a second orthogonal platform, qPCR. Accordingly, we designed a custom qPCR copy-number array containing a set of 11 genes, each representing a distinct genomic locus that showed recurrent sCNAs (frequency ≥ 30 %) among the 30 AA CRC cases in the WES dataset, as detected by ENVE (Additional file 1: Table S2). Using this qPCR array, we estimated sCNAs in a subset of AA CRC cases (N = 6), where each of the cancers showed copy-number alteration in at least one of the 11 genes (Fig. 6a). Of note, these six cases were not represented in the 12 samples used for the SNP array analysis, thus allowing for an independent evaluation of ENVE’s performance. Respective matched normal samples from these six cancers, along with an additional six AA normal samples, were used as diploid genome controls in the qPCR analysis. We again used the WES-based sCNA calls from Control-FREEC for these six CRC cases as an additional key comparator in this analysis. Comparison of amplifications, deletions, and copy-neutral calls between qPCR and ENVE showed a significantly higher overall concordance of 72.72 % (Chi-square *P* = 0.049, Fig. 6b) compared to the 59 % concordance observed between qPCR and Control-FREEC (Additional file 2: Figure S7). Notably, 54 % of the qPCR and ENVE concordant alterations exhibited low tumor/normal LogRatios ranging between −1 and 0.7 in the WES data (Fig. 6a), likely suggesting that the higher performance of ENVE compared to Control-FREEC results from ENVE’s ability to detect sCNAs even at low tumor/normal read depth ratios in these stromal-admixed tumor samples. It is important to note that although the genes and samples selected for this comparison were chosen based on ENVE’s output, we had no a priori expectation of the qPCR results, thus allowing for a fair comparison with Control-FREEC.

Taken together, although neither qPCR nor SNP arrays are gold-standard techniques for a formal evaluation of the sensitivity and specificity of ENVE and Control-FREEC, our comparative analyses based on these commonly used techniques underscore the ability of the ENVE methodology to reliably detect sCNAs in variable stromal admixture tumor tissues, without having to resort to complex and unstable estimations of tumor content or ploidy.

### Characterization of sCNA landscapes in AA CRCs

**Fig. 7:**
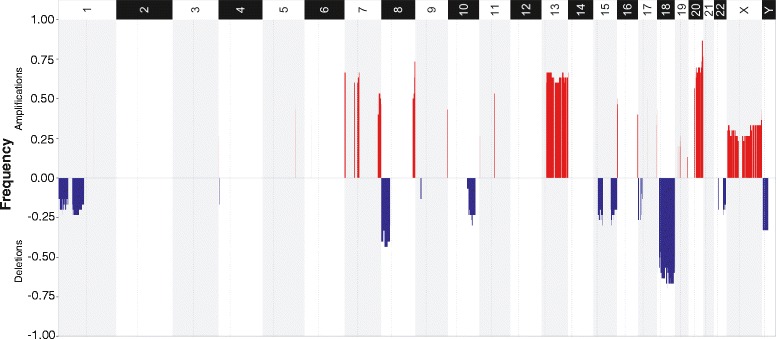
Recurrent somatic copy-number alterations in AA CRCs. GISTIC plot showing the frequencies (Y-axis) of significant recurrent amplifications (*red*) and deletions (*blue*), as identified by ENVE, in the 30 AA CRC cases. Recurrent arm-level alterations are observed within autosomal regions, including deletions in 1p, 8p, 15q, 18p, and 18q, and amplifications in 7p, 8q, 13q, and 20q

Using the ENVE-significant alterations as input (Additional file 1: Table S2), we next identified chromosomal regions showing significant (q-value ≤ 0.25) recurrent focal and arm-level alterations in AA CRCs using the GISTIC tool [27] (see “Methods”; Additional file 1: Tables S4–S6, Fig. 7). While focal sCNAs occurred throughout the length of respective chromosomes (Additional file 1: Tables S4 and S5), GISTIC’s broad-level analysis showed significant (q-value ≤ 0.25) chromosomal arm-level deletions specifically in 1p, 8p, 14q, 15q, 18p, and 18q, and amplifications in 1q, 7p, 8q, 13q, 19q, 20p, and 20q in AA CRCs (Additional file 1: Table S6). Furthermore, chromosomal regions containing well-known CRC tumor suppressor genes (*TP53*, *DCC*, *SMAD4*, *SMAD2*) [9, 35, 36] showed recurrent copy-number deletions in ≥25 % of AA CRC cases. Conversely, copy-number amplifications in 13q and 20q loci, regions known to harbor candidate oncogenes [37–39], were observed in ≥27 % of AA CRC cases.

We next asked if there were any recurrent sCNA signatures identified in AA CRCs (Fig. 7) that were significantly different from Caucasian CRCs. Accordingly, we identified a set of 30 predominantly late-stage MSS Caucasian CRC cases from the TCGA WES cohort (Additional file 1: Table S1), and evaluated for significant sCNAs (ENVE *P* ≤ 0.05; Additional file 1: Table S3) followed by GISTIC analysis to identify recurrent chromosomal arm-level alterations (q ≤ 0.25; Additional file 1: Table S6). Assessing for significant chromosomal arm-level alterations in these two cohorts, however, showed no marked differences in their frequencies between the AA and TCGA CRC WES datasets, and/or between the AA CRC WES and the TCGA CRC SNP array datasets (Additional file 1: Table S6).

While the majority of the recurrent sCNAs observed in AA CRCs (Fig. 7) were consistent with those previously reported for colon cancers [9], there is a likelihood that ethnicity-associated differences exist in both the location and frequency of focal sCNAs in CRCs. Although we identified a significant number of focal copy-number alterations in AA CRCs (Additional file 1: Table S5), a larger platform-matched and algorithm-matched sCNA analysis is necessary to systematically characterize ethnicity-specific differences in focal sCNAs in CRCs. Nonetheless, our prior study detailing the gene-mutational landscapes in AA CRCs [20], together with our current comprehensive characterization of sCNA landscapes in AA CRCs, uncovers recurrent genetic aberrations that are potentially associated with CRC development in the AA population.

## Discussion

We have developed a robust and unbiased method for detecting somatic copy-number alterations using WES data. Performance evaluation of ENVE in two independent WES tumor tissue datasets showed a high concordance between ENVE and SNP array and qPCR-based sCNA estimates (Figs. 5 and 6). In addition, we found ENVE significantly and consistently outperformed the best-in-class published WES-based sCNA detection algorithm [19], Control-FREEC [12] (Figs. 5 and 6, Additional file 2: Figures S4–S7). More importantly, our performance evaluations strongly indicate that ENVE has high sensitivity and specificity in detecting sCNAs from WES data derived from stromal-admixed tumor samples. In particular, our comparative analyses reveal the effectiveness of ENVE in detecting genuine sCNAs even at low tumor/normal segmental LogRatios (−1 to 0.7) (Fig. 6a), strongly underscoring the drawbacks with using pre-defined LogRatio value cutoffs to identify sCNAs in tumors. In fact, examination of the relationship between the LogRatios of individual segments and ENVE-based *P*-values (Additional file 2: Figure S8) shows that no single segmental LogRatio-cutoff value would have captured all recurrent copy-number amplifications or deletions in either the AA or TCGA CRC WES datasets. Besides, the commonly observed variability in cancer cell content among clinical specimens would preclude the use of a single LogRatio-cutoff value for determining recurrent sCNAs. Although some published copy-number algorithms have attempted to overcome the challenge of defining LogRatio-cutoffs by inferring the tumor content and ploidy of each sample to estimate the absolute tumor copy-number [16, 18, 40, 41], these estimates are often unstable, with these algorithms differing in their underlying assumptions, which may not always correspond to the complex chromosomal architecture in tumors [42]. Our approach, in contrast, does not infer tumor content or ploidy, but provides a probabilistic estimate of the presence of sCNAs in tumors given the inherent noise in WES measurements as estimated from non-malignant normal diploid samples, and therefore offers a simpler and robust alternative.

Because one of the key characteristics of ENVE is the use of normal diploid samples for capturing inherent noise associated with WES data, we used DNA samples derived from 54 immortalized lymphoblastoid cell lines established from patients’ peripheral blood lymphocytes to determine whether noise threshold estimates are sensitive to the number of normal diploid samples used for noise assessment. We estimated chromosome-specific noise thresholds using normal–normal comparisons derived from random groups of 16–54 samples in increments of 2, repeated ten times. We found the ENVE estimates of noise thresholds across chromosomes to be nearly all stable with respect to the number of diploid samples used for the estimation (Additional file 2: Figure S9). Although the chromosome-specific noise thresholds are not sensitive to the number of diploid samples being used, reliable estimation of the parameters of the GEV distribution requires 100–150 extreme values [43], corresponding to a lower acceptable limit of 15–20 normal diploid samples. Therefore, we suggest that using 15–20 normal samples is sufficient to model the inherent noise in WES data, and as such is computationally efficient. We therefore anticipate that ENVE’s key feature involving modeling of inherent noise in WES data will enable its broad application across studies, where population-matched and platform-matched normal diploid DNA samples are frequently available.

Foreseeing a likely practical situation where a normal sample matching the tumor may not be available from the patient, we further evaluated the performance of ENVE in a simulated circumstance where each of the tumor samples was compared to a pooled set of normal samples derived from the WES data. This analysis was performed using computationally derived pooled normal samples for both the AA CRC WES and SNP array datasets (see “Methods”). Next, we compared the ENVE sCNA calls with SNP array-based sCNA calls in the same 12 AA CRC cases from above (Fig. 5). Notably, in this pooled analysis (Additional file 2: Figure S10), ENVE exhibited high concordance rates with the SNP array calls for both amplifications (97.72 %) and deletions (92.86 %), as compared to the matched normal analysis, further suggesting that ENVE remains a viable methodology for reliably detecting sCNAs in tumors even in the absence of a matched normal sample.

We note that one of the limitations of published algorithms [12–18] is their exclusive applicability to deep-sequencing data derived from fresh-frozen material but not archived formalin-fixed paraffin-embedded (FFPE) biospecimens. While sequencing of archived FFPE DNA allows for de novo characterization of gene mutations, as shown by us and others [11, 44], estimation of copy-number alterations using WES of FFPE specimens remains challenging owing to poor DNA quality in archived FFPE tumor samples. This, in turn, may result in enhanced inherent noise, which may also be prevalent in FFPE-derived normal diploid DNA samples. We have not assessed the performance of ENVE in archival FFPE samples, but we anticipate that ENVE’s noise-modeling feature may reliably capture the degree of inherent noise in FFPE samples, thus potentially enabling use of the extensive clinically annotated tumor samples held in pathology archives.

One potential limitation of ENVE is that, while it models sources of inherent noise in WES data, it does not explicitly model the likely occurrences of genomic complexities, such as aneuploidy and hyperploidy, in the tumors. This may possibly influence the true positive/negative sCNA detection rates of the current ENVE framework. However, estimating allele frequencies in addition to LogRatios from WES data is a conceivable extension to the current ENVE framework, and may address the influence of such aberrations. Another likely limitation of ENVE is the requirement of at least 15–20 platform-matched normal samples in order to capture and model the inherent noise in WES data. However, because most cancer-profiling studies are designed to include the collection of platform-matched normal samples (matched/unmatched with the tumors), this limitation is likely not burdensome. More importantly, we note that the ENVE’s unique noise-modeling feature, not included in any of the other published sCNA detection algorithms, provides detailed and otherwise unavailable comprehension of the inherent noise in any given WES dataset to the user (Figs. 1b,c and 2b,c), thus allowing for reliable interrogation of sCNAs in the tumor samples in a platform-agnostic manner.

## Conclusions

We present ENVE as a robust method for detecting sCNAs in WES-based studies using either matched or unmatched tumor/normal samples, without the need for complex parameter choices or extensive user intervention. In particular, ENVE reliably detects sCNAs in stromal-admixed tumor samples and is therefore expected to be broadly applicable across cancer-profiling studies. We believe this user-friendly methodology should be portable to any massively parallel DNA sequencing platform.

## Additional files

Additional file 1: Table S1. Cohorts, ethnicity, and tumor stage of samples used for WES, SNP array, and qPCR. **Table S2.** Regions with significant copy-number alterations, as detected by ENVE, in the 30 AA CRC WES cases. **Table S3.** Regions with significant copy-number alterations, as detected by ENVE, in the 30 predominantly late-stage Caucasian TCGA CRC WES cases. **Table S4.** Recurrent somatic copy-number altered regions in the 30 AA CRC WES cases estimated by GISTIC. **Table S5.** Recurrent focal copy-number amplifications and deletions in the 30 AA CRC WES cases estimated by GISTIC. **Table S6.** Chromosomal arm-level sCNA frequencies in AA and TCGA CRCs estimated by GISTIC. (XLSX 535 kb) Additional file 2: Figure S1. Fraction of chromosomal coverage across segmental LogRatio thresholds in AA normal–normal comparisons. **Figure S2.** Entropy of chromosomal coverage across segmental LogRatio thresholds in AA normal–normal comparisons. **Figure S3**. Concordance assessment of ENVE/Control-FREEC sCNA segments with SNP array. **Figure S4.** Performance evaluation of Control-FREEC with contamination-correction against ENVE. **Figure S5**. Performance evaluation of ENVE against Control-FREEC across SNP array Segment-Mean cutoffs in the TCGA dataset. **Figure S6**. Impact of sequencing read depth on ENVE versus Control-FREEC performance in the TCGA WES dataset. **Figure S7.** Concordance analysis of Control-FREEC-based and qPCR-based sCNA estimates. **Figure S8.** Relationship between the tumor/normal Segmental LogRatios and ENVE *P-*value. **Figure S9.** Effect of the number of normal samples on ENVE noise-threshold estimates. **Figure S10.** Performance evaluation of ENVE in matched- versus pooled normal analysis scenarios. (PDF 2411 kb)

## Abbreviations

AA: African American
BAM: binary sequence alignment/map
CNV: copy-number variation
CRC: colorectal cancer
DCC: DCC netrin 1 receptor
ENVE: Extreme value distribution based somatic copy-Number Variation Estimation
FFPE: formalin-fixed paraffin-embedded
GEV: generalized extreme value distribution
MSS: microsatellite stable
qPCR: quantitative real-time polymerase chain reaction
sCNA: somatic copy-number alteration
SMAD2: SMAD family member 2
SMAD4: SMAD family member 4
SNP: single nucleotide polymorphism
TCGA: The Cancer Genome Atlas
TP53: tumor protein p53
WES: whole-exome sequencing

## References

[1] Al-Kuraya K, Schraml P, Torhorst J, Tapia C, Zaharieva B, Novotny H (2004). Prognostic relevance of gene amplifications and coamplifications in breast cancer. Cancer Res..

[2] Zack TI, Schumacher SE, Carter SL, Cherniack AD, Saksena G, Tabak B (2013). Pan-cancer patterns of somatic copy number alteration. Nat Genet..

[3] Ocak S, Yamashita H, Udyavar AR, Miller AN, Gonzalez AL, Zou Y (2010). DNA copy number aberrations in small-cell lung cancer reveal activation of the focal adhesion pathway. Oncogene..

[4] Hieronymus H, Schultz N, Gopalan A, Carver BS, Chang MT, Xiao Y (2014). Copy number alteration burden predicts prostate cancer relapse. Proc Natl Acad Sci U S A..

[5] Pinkel D, Segraves R, Sudar D, Clark S, Poole I, Kowbel D (1998). High resolution analysis of DNA copy number variation using comparative genomic hybridization to microarrays. Nat Genet..

[6] Lucito R, Healy J, Alexander J, Reiner A, Esposito D, Chi M (2003). Representational oligonucleotide microarray analysis: a high-resolution method to detect genome copy number variation. Genome Res..

[7] Bignell GR, Huang J, Greshock J, Watt S, Butler A, West S (2004). High-resolution analysis of DNA copy number using oligonucleotide microarrays. Genome Res..

[8] Stransky N, Egloff AM, Tward AD, Kostic AD, Cibulskis K, Sivachenko A (2011). The mutational landscape of head and neck squamous cell carcinoma. Science..

[9] Cancer Genome Atlas Network (2012). Comprehensive molecular characterization of human colon and rectal cancer. Nature..

[10] Kamalakaran S, Varadan V, Janevski A, Banerjee N, Tuck D, McCombie WR (2013). Translating next generation sequencing to practice: opportunities and necessary steps. Mol Oncol..

[11] Van Allen EM, Wagle N, Stojanov P, Perrin DL, Cibulskis K, Marlow S (2014). Whole-exome sequencing and clinical interpretation of formalin-fixed, paraffin-embedded tumor samples to guide precision cancer medicine. Nat Med..

[12] Boeva V, Popova T, Bleakley K, Chiche P, Cappo J, Schleiermacher G (2012). Control-FREEC: a tool for assessing copy number and allelic content using next-generation sequencing data. Bioinformatics..

[13] Chiang DY, Getz G, Jaffe DB, O’Kelly MJ, Zhao X, Carter SL (2009). High-resolution mapping of copy-number alterations with massively parallel sequencing. Nat Methods..

[14] Koboldt DC, Zhang Q, Larson DE, Shen D, McLellan MD, Lin L (2012). VarScan 2: somatic mutation and copy number alteration discovery in cancer by exome sequencing. Genome Res..

[15] Ha G, Roth A, Lai D, Bashashati A, Ding J, Goya R (2012). Integrative analysis of genome-wide loss of heterozygosity and monoallelic expression at nucleotide resolution reveals disrupted pathways in triple-negative breast cancer. Genome Res..

[16] Gusnanto A, Wood HM, Pawitan Y, Rabbitts P, Berri S (2012). Correcting for cancer genome size and tumour cell content enables better estimation of copy number alterations from next-generation sequence data. Bioinformatics..

[17] Krishnan NM, Gaur P, Chaudhary R, Rao AA, Panda B (2012). COPS: a sensitive and accurate tool for detecting somatic copy number alterations using short-read sequence data from paired samples. PLoS One..

[18] Bao L, Pu M, Messer K (2014). AbsCN-seq: a statistical method to estimate tumor purity, ploidy and absolute copy numbers from next-generation sequencing data. Bioinformatics.

[19] Alkodsi A, Louhimo R, Hautaniemi S (2014). Comparative analysis of methods for identifying somatic copy number alterations from deep sequencing data. Briefings Bioinformatics..

[20] Guda K, Veigl ML, Varadan V, Nosrati A, Ravi L, Lutterbaugh J (2015). Novel recurrently mutated genes in African American colon cancers. Proc Nat Acad Sci U S A..

[21] Umar A, Risinger JI, Hawk ET, Barrett JC (2004). Testing guidelines for hereditary non-polyposis colorectal cancer. Nat Rev Cancer..

[22] Li H. Burrows-Wheeler Aligner. http://sourceforge.net/projects/bio-bwa/. Accessed 21 Jan 2014.

[23] Li H, Durbin R (2009). Fast and accurate short read alignment with Burrows-Wheeler transform. Bioinformatics..

[24] Broad Institute: Picard Tools. http://broadinstitute.github.io/picard/. Accessed 21 Jan 2014.

[25] Cancer Genomics Hub. https://cghub.ucsc.edu. Accessed 09 Jan 2015.

[26] The Cancer Genome Atlas. https://tcga-data.nci.nih.gov/tcga/. Accessed 09 Jan 2015.

[27] Mermel CH, Schumacher SE, Hill B, Meyerson ML, Beroukhim R, Getz G (2011). GISTIC2.0 facilitates sensitive and confident localization of the targets of focal somatic copy-number alteration in human cancers. Genome Biol..

[28] SABiosciences: qBiomarker Data Analysis version 1.2. http://pcrdataanalysis.sabiosciences.com/cnv/CNVanalysis.php. Accessed 08 Apr 2014.

[29] ENVE Tool. https://github.com/ENVE-Tools/ENVE.

[30] Olshen AB, Venkatraman ES, Lucito R, Wigler M (2004). Circular binary segmentation for the analysis of array-based DNA copy number data. Biostatistics..

[31] Rousseeuw PJ. Silhouettes: a graphical aid to the interpretation and validation of cluster analysis. J Comput Appl Math. 1986;20:53–65. doi:10.1016/0377-0427(87)90125-7.

[32] Coles S (2001). An introduction to statistical modeling of extreme values. Springer series in statistics.

[33] Li A, Liu Z, Lezon-Geyda K, Sarkar S, Lannin D, Schulz V (2011). GPHMM: an integrated hidden Markov model for identification of copy number alteration and loss of heterozygosity in complex tumor samples using whole genome SNP arrays. Nucleic Acids Res..

[34] Yu Z, Liu Y, Shen Y, Wang M, Li A (2014). CLImAT: accurate detection of copy number alteration and loss of heterozygosity in impure and aneuploid tumor samples using whole-genome sequencing data. Bioinformatics..

[35] Wood LD, Parsons DW, Jones S, Lin J, Sjoblom T, Leary RJ (2007). The genomic landscapes of human breast and colorectal cancers. Science..

[36] Sjoblom T, Jones S, Wood LD, Parsons DW, Lin J, Barber TD (2006). The consensus coding sequences of human breast and colorectal cancers. Science..

[37] Day E, Poulogiannis G, McCaughan F, Mulholland S, Arends MJ, Ibrahim AE (2013). IRS2 is a candidate driver oncogene on 13q34 in colorectal cancer. Int J Exp Pathol..

[38] Tabach Y, Kogan-Sakin I, Buganim Y, Solomon H, Goldfinger N, Hovland R (2011). Amplification of the 20q chromosomal arm occurs early in tumorigenic transformation and may initiate cancer. PLoS One..

[39] Brim H, Lee E, Abu-Asab MS, Chaouchi M, Razjouyan H, Namin H (2012). Genomic aberrations in an African American colorectal cancer cohort reveals a MSI-specific profile and chromosome X amplification in male patients. PLoS One..

[40] Carter SL, Cibulskis K, Helman E, McKenna A, Shen H, Zack T (2012). Absolute quantification of somatic DNA alterations in human cancer. Nat Biotechnol..

[41] Van Loo P, Nordgard SH, Lingjaerde OC, Russnes HG, Rye IH, Sun W (2010). Allele-specific copy number analysis of tumors. Proc Natl Acad Sci U S A..

[42] Lonnstedt IM, Caramia F, Li J, Fumagalli D, Salgado R, Rowan A (2014). Deciphering clonality in aneuploid breast tumors using SNP array and sequencing data. Genome Biol..

[43] Hosking JRM, Wallis JR, Wood EF (1985). Estimation of the generalized extreme-value distribution by the method of probability-weighted moments. Technometrics..

[44] Adams MD, Veigl ML, Wang Z, Molyneux N, Sun S, Guda K (2012). Global mutational profiling of formalin-fixed human colon cancers from a pathology archive. Modern Pathol..

