# Supplementary material for: ENVE: a novel computational framework characterizes copy-number mutational landscapes in colorectal cancers from African American patients
Source: Genome Med. 2015 Jul 20;7(1):69. doi: 10.1186/s13073-015-0192-9 (PMC4534088; doi:10.1186/s13073-015-0192-9)
Supplement: Additional file 2: Figure S1. — Fraction of chromosomal coverage across segmental LogRatio thresholds in AA normal–normal comparisons. Figure S2. Entropy of chromosomal coverage across segmental LogRatio thresholds in AA normal–normal comparisons. Figure S3. Concordance assessment of ENVE/Control-FREEC sCNA segments with SNP array. Figure S4. Performance evaluation of Control-FREEC with contamination-correction against ENVE. Figure S5. Performance evaluation of ENVE against Control-FREEC across SNP array Segment-Mean cutoffs in the TCGA dataset. Figure S6. Impact of sequencing read depth on ENVE versus Control-FREEC performance in the TCGA WES dataset. Figure S7. Concordance analysis of Control-FREEC-based and qPCR-based sCNA estimates. Figure S8. Relationship between the tumor/normal Segmental LogRatios and ENVE P-value. Figure S9. Effect of the number of normal samples on ENVE noise-threshold estimates. Figure S10. Performance evaluation of ENVE in matched- versus pooled normal analysis scenarios. (PDF 2411 kb) [file 13073_2015_192_MOESM2_ESM.pdf]

Supplementary Figures

ENVE: A novel computational framework characterizes copy-number mutational landscapes in African American colon cancers

Vinay Varadan, Salendra Singh, Arman Nosrati, Lakshmeswari Ravi, James Lutterbaugh, Jill S. Barnholtz-Sloan, Sanford D. Markowitz, Joseph E. Willis, and Kishore Guda

Table of Contents:

Figure S1. Fraction of chromosomal coverage across segmental LogRatio thresholds in AA normal-normal comparisons.

Figure S2. Entropy of chromosomal coverage across segmental LogRatio thresholds in AA normal-normal comparisons.

Figure S3. Concordance assessment of ENVE/Control-FREEC sCNA segments with SNP-array.

Figure S4. Performance evaluation of Control-FREEC with contamination correction against ENVE.

Figure S5. Performance evaluation of ENVE against Control-FREEC across SNP-array Segment-Mean cutoffs in the TCGA dataset.

Figure S6. Impact of sequencing read-depth on ENVE versus Control-FREEC performance in the TCGA WES dataset.

Figure S7. Concordance analysis of Control-FREEC- and qPCR-based sCNA estimates.

Figure S8. Relationship between the tumor/normal Segmental LogRatios and ENVE Pvalue.

Figure S9. Effect of the number of normal samples on ENVE noise-threshold estimates.

Figure S10. Performance evaluation of ENVE in matched- versus pooled-normal analysis scenarios.

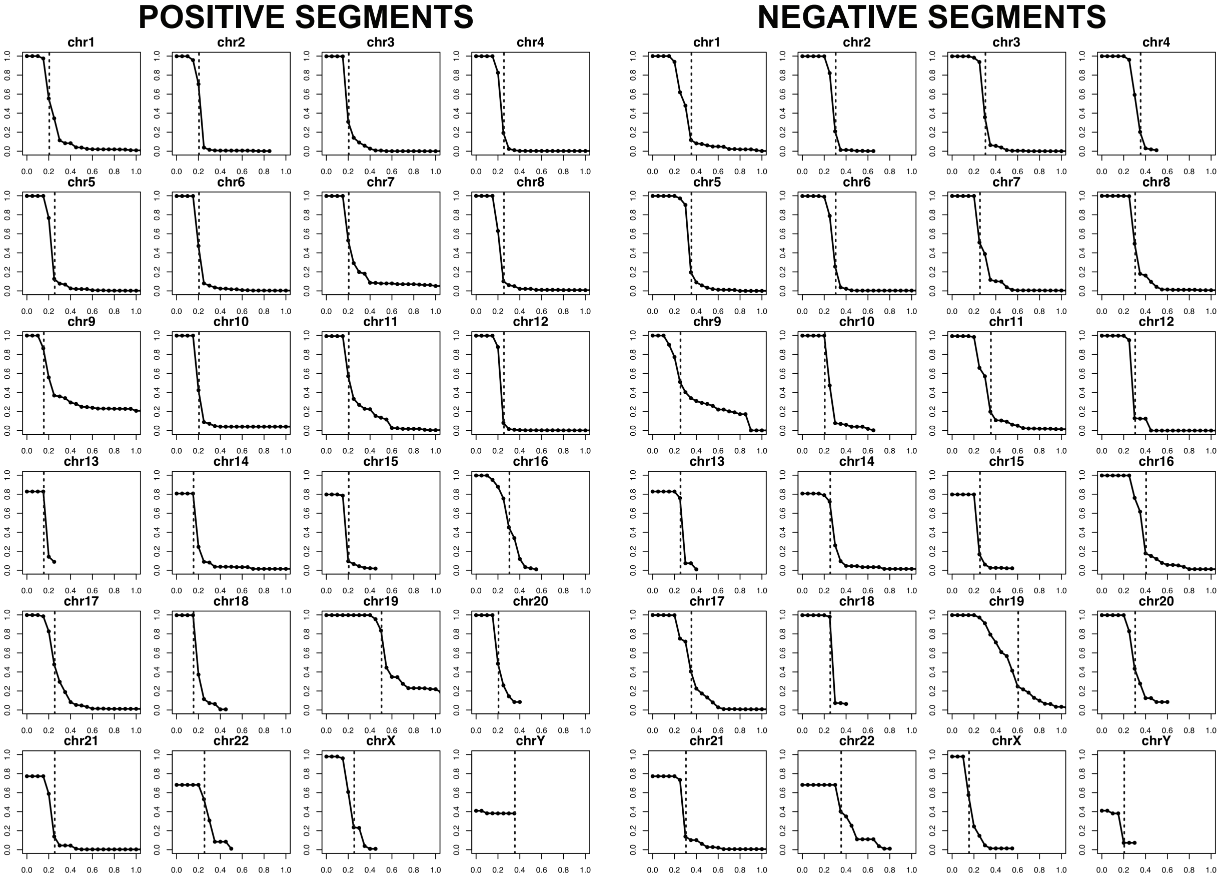

Figure S1. **Fraction of chromosomal coverage across segmental LogRatio thresholds in AA normal-normal comparisons.** The fraction of chromosomal coverage (y-axis) is plotted against the minimal segmental LogRatio of copy-number altered segments (x-axis), for segments exhibiting both positive and negative LogRatios within 435 normal-normal comparisons derived from 30 normal diploid AA exomes. The vertical dotted line within each chromosome plot corresponds to the LogRatio threshold at which the largest drop in chromosomal coverage is observed.

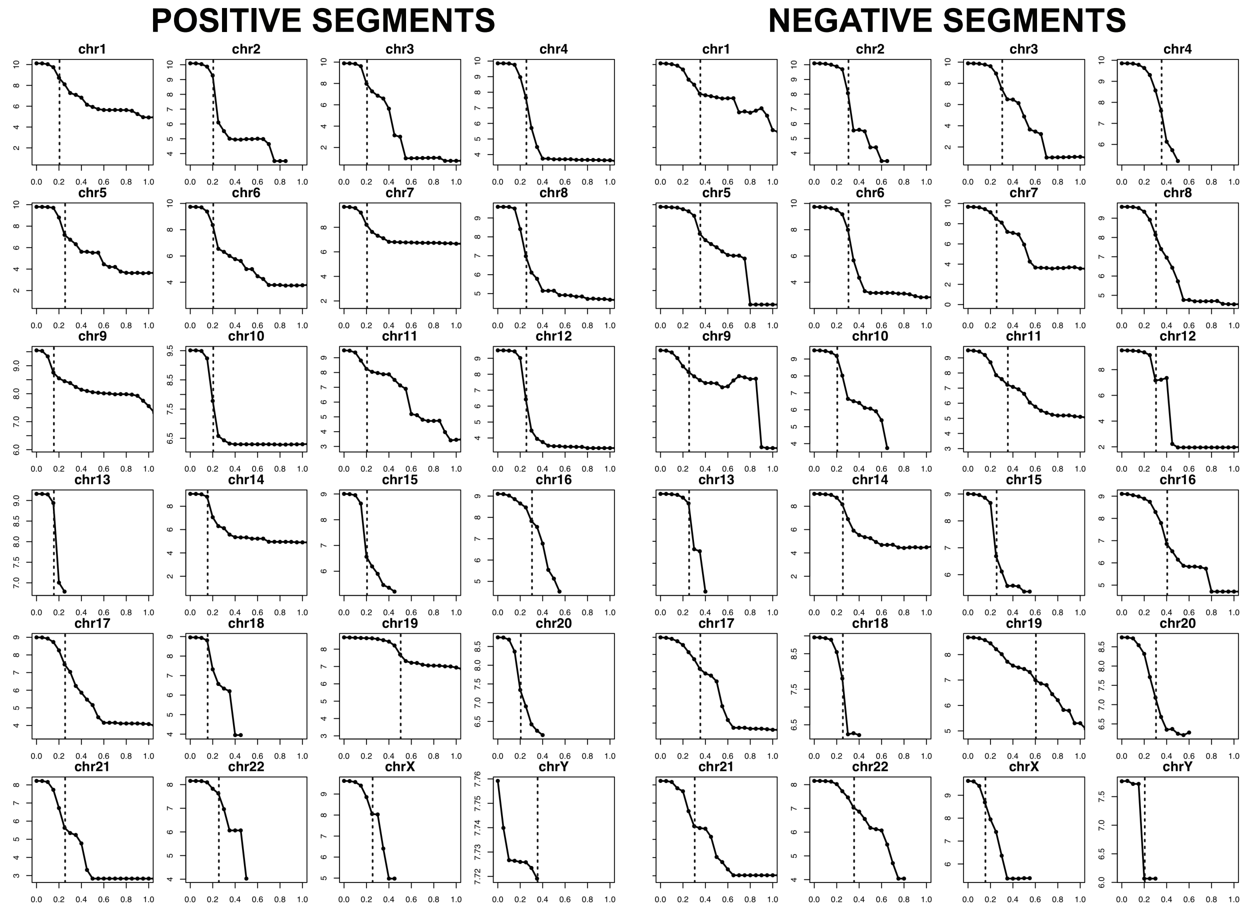

Figure S2. **Entropy of chromosomal coverage across segmental LogRatio thresholds in AA normal-normal comparisons.** The entropy of chromosome coverage (y-axis) is plotted against the minimal segmental LogRatio of copy-number altered segments (x-axis), for segments exhibiting both positive and negative LogRatios within the 435 normal-normal comparisons derived from 30 normal diploid AA exomes. The vertical dotted line within each chromosome plot corresponds to the LogRatio threshold at which the largest drop in chromosomal coverage is observed (from Fig. S2).

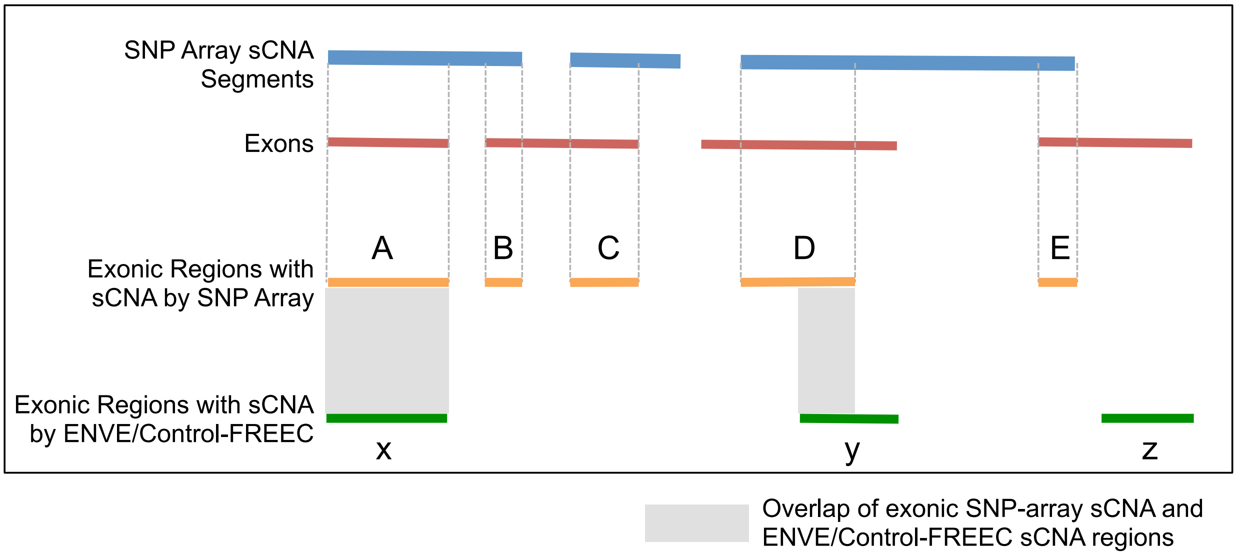

$$\text{Percent Concordance} = {100 * (x + y)}/{(A + B + C + D + E)}$$

Figure S3. **Concordance assessment of ENVE/Control-FREEC sCNA segments with SNP-array.** Given the known disparities in the genomic coverage and lengths of copy-number altered segment between SNP-arrays and whole exomes, we first identified all the SNP-array sCNA regions that fall within exons with at least 1 basepair overlap. We then identified all the sCNA exonic regions called by the respective WES algorithms. As shown in the figure, the percent concordance was accordingly calculated as the ratio of the total length of all concordant exonic sCNA regions called by ENVE or Control-FREEC to the total length of the exonic SNP-array sCNA regions. Exonic sCNA regions called by the respective WES algorithms that were concordant with exonic SNP-array sCNA regions showed at least 75% sequence overlap.

A

Dataset	Performance Estimates	Amplifications		Deletions	
		ENVE	Control FREEC (Cont_Cor)	ENVE	Control FREEC (Cont_Cor)
AA CRC	Median Genes Altered	3563	7662	3490	1190
	% Concordance with SNP Array sCNA Calls	***97.32***	***90.11***	***90.6***	***47.65***
TCGA	Median Genes Altered	1950	4134	1478	3259
	% Concordance with SNP Array sCNA Calls	***97.68***	***89.47***	***78.22***	***83.96***

B

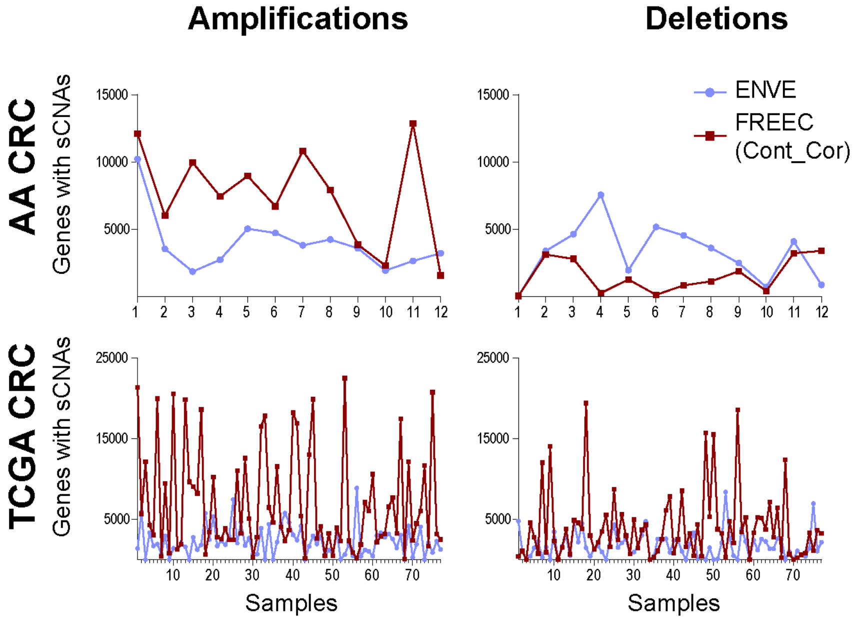

Figure S4. **Performance evaluation of Control-FREEC with contamination correction against ENVE.** Panel A) shows the median number of genes with sCNAs in WES data, as detected by Control-FREEC run in the contamination correction mode and ENVE, along with their median percent concordance with SNP-array based sCNA calls. Panel B) shows the number of genes with sCNAs (Y-axis) in each sample (X-axis), as detected by ENVE (blue) and Control-FREEC (red), in AA and TCGA CRC WES datasets. For AA CRC significant sCNAs as detected by the Partek Suite were used for comparison, while for TCGA CRC SNP-array segments with Segment-Mean cutoffs of ±0.5 were used for comparison. Cont_Cor indicates contamination correction.

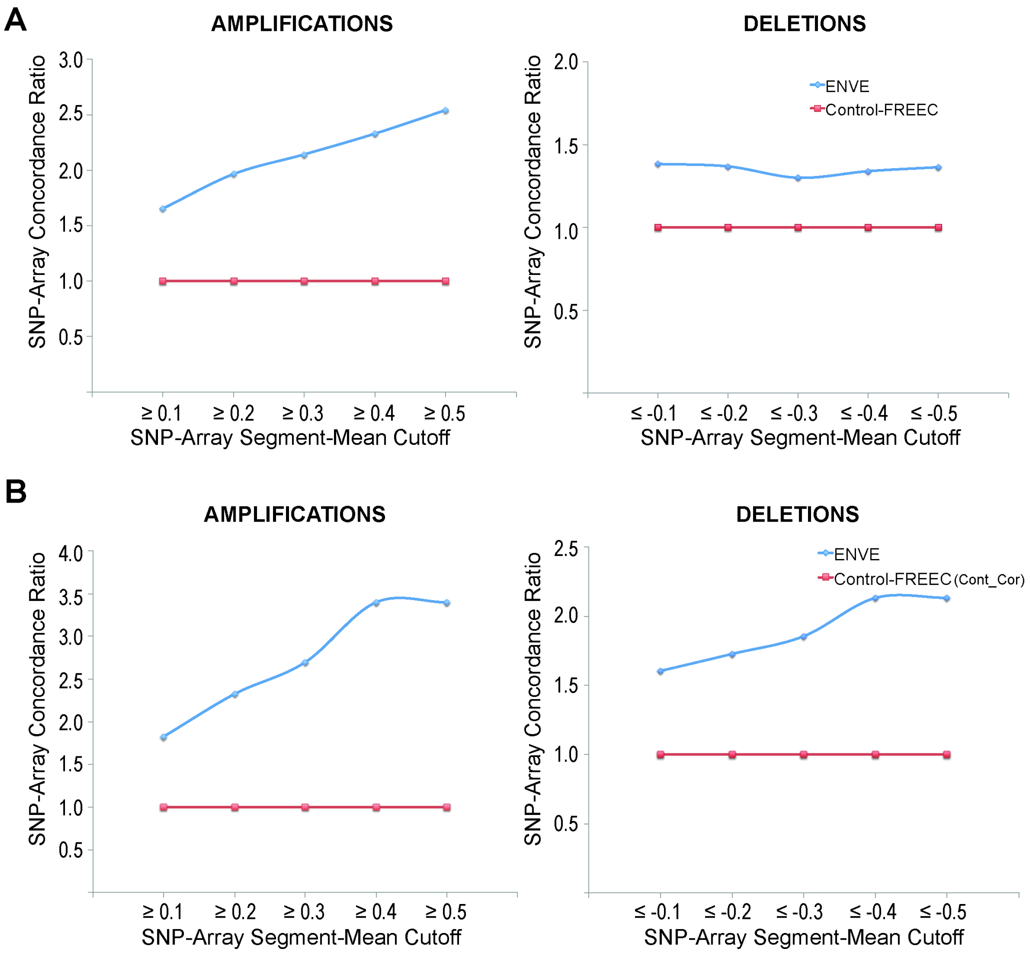

Figure S5. **Performance evaluation of ENVE against Control-FREEC across SNP-array Segment-Mean cutoffs in the TCGA dataset.** For this comparison, the average concordance of ENVE- or Control-FREEC-based sCNA calls with the SNP-array calls was estimated at each of the five SNP-array Segment-Mean cutoff values (X-axis). To account for inherent differences in the specificity of ENVE versus Control-FREEC algorithms, the average concordance values were further normalized to the total number of genes showing sCNAs, as detected by the respective WES algorithm, in the 77 TCGA tumors. The Y-axis shows the ratio (ENVE vs. Control-FREEC) of the respective normalized SNP-array concordance, defined as:

$$(ENVE_{Concordance}/ENVE_{\#GenesAltered})/(FREEC_{Concordance}/FREEC_{\#GenesAltered})$$

across the different SNP-array Segment-Mean cutoffs (X-axis), with Control-FREEC as the baseline. Panel A) shows the comparison with Control-FREEC run in the default WES mode. Panel B) shows the comparison with Control-FREEC run in the contamination correction mode (Cont_Cor). Note that ENVE consistently shows higher SNP-array concordance as compared to Control-FREEC, for both copy-number amplifications and deletions, across all comparisons and SNP-array Segment-Mean cutoff values.

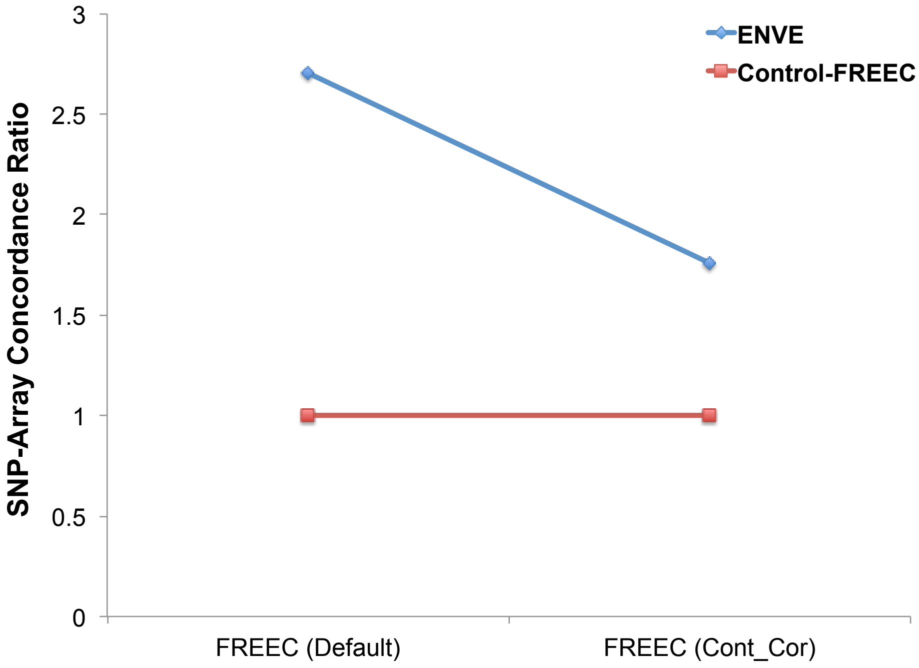

Figure S6. **Impact of sequencing read-depth on ENVE versus Control-FREEC performance in the TCGA WES dataset.** For this comparison, we first down-sampled the number of reads by 50% in a random set of 30 normal and tumor sample-pairs from the TCGA WES dataset. We then characterized sCNA profiles using ENVE and Control-FREEC on this down-sampled dataset. We estimated the average concordance of ENVE- or Control-FREEC-based sCNA calls with the SNP-array calls using a SNP-array Segment-Mean cutoff value of 0.5. Similar to Figure S5, the average concordance values were further normalized to the total number of genes showing sCNAs (amplifications+deletions) to account for inherent differences in the specificity of the algorithms. Plotted are the ratios (ENVE vs. Control-FREEC) of the respective normalized SNP-array concordance (Y-axis), with Control-FREEC run in the default WES and in the contamination correction mode (Cont_Cor) (X-axis). Note that ENVE consistently shows higher SNP-array concordance in this down-sampled dataset as compared to Control-FREEC.

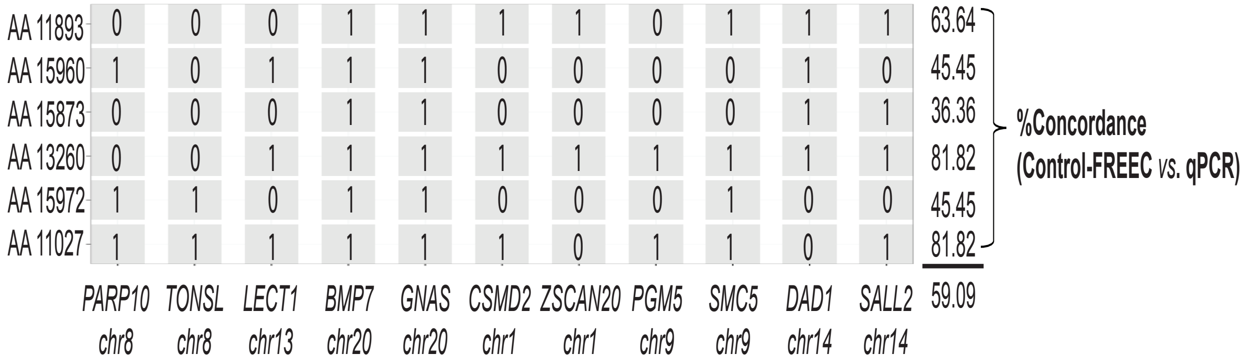

Figure S7. **Concordance analysis of Control-FREEC- and qPCR-based sCNA estimates.** Matrix showing concordance (1) and discordance (0) between Control-FREEC and qPCR CN estimates for the 11 genes in each of the 6 AA CRC cases. For each case, genes showing no CN alterations by both Control-FREEC and qPCR analyses, as well as genes showing significant CN alterations in the same direction by both platforms were deemed concordant. The resulting percent concordance between Control-FREEC- and qPCR-based CN estimates in each sample is shown on the right, with an overall concordance rate of 59%.

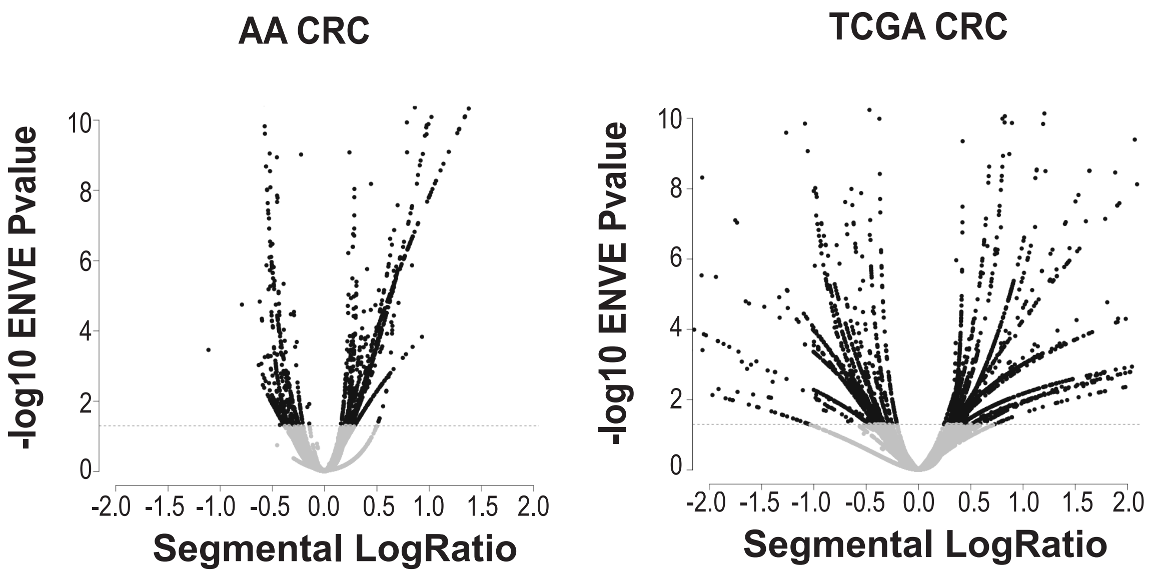

Figure S8. Relationship between the tumor/normal Segmental LogRatios and ENVE Pvalue. The LogRatios of the individual segments (X-axis) and the ENVE-based Pvalues (Y-axis) are plotted for both the significant (ENVE Pvalue ≤0.05; solid black points) and non-significant (grey points) segments in the AA CRC and TCGA CRC WES datasets. The significance threshold (ENVE Pvalue =0.05) is indicated by the horizontal dotted grey line in each plot.

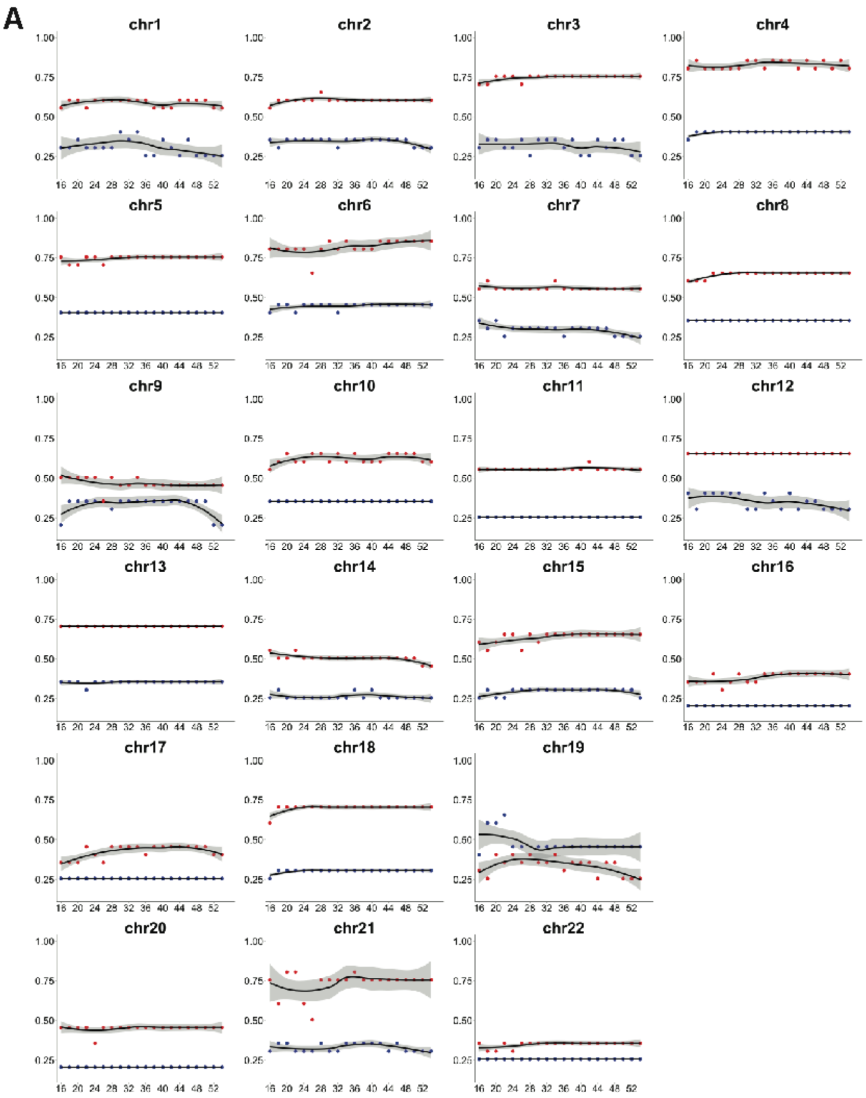

Figure S9. **Effect of the number of normal samples on ENVE noise-threshold estimates. A)** We evaluated the effect of the number of normal-normal comparisons on noise-threshold estimates using a whole-exome sequencing dataset consisting of DNA samples derived from 54 immortalized lymphoblastoid cell lines established from patient peripheral blood lymphocytes. Noise-thresholds (Y-axis) were estimated using normal-normal comparisons derived from random groups of 16 samples to 54 samples in increments of 2 (X-axis), repeated 10 times. The blue and the red points represent the maximum positive and negative noise-thresholds for the 10 random repeats, respectively, across each of the random sample groups. The grey area represents the 95% confidence interval for the best-fitting line (black) across the sample groups using Loess smoothing. B) Modeling of inherent noise using a random set of 30 normal samples derived from this dataset. Plots show chromosomal coverage by copy-number altered segments at different LogRatio thresholds across all chromosomes for the positive (A) and negative (B) LogRatio deviations. Bold horizontal lines within each chromosome indicate the noise-threshold values.

B

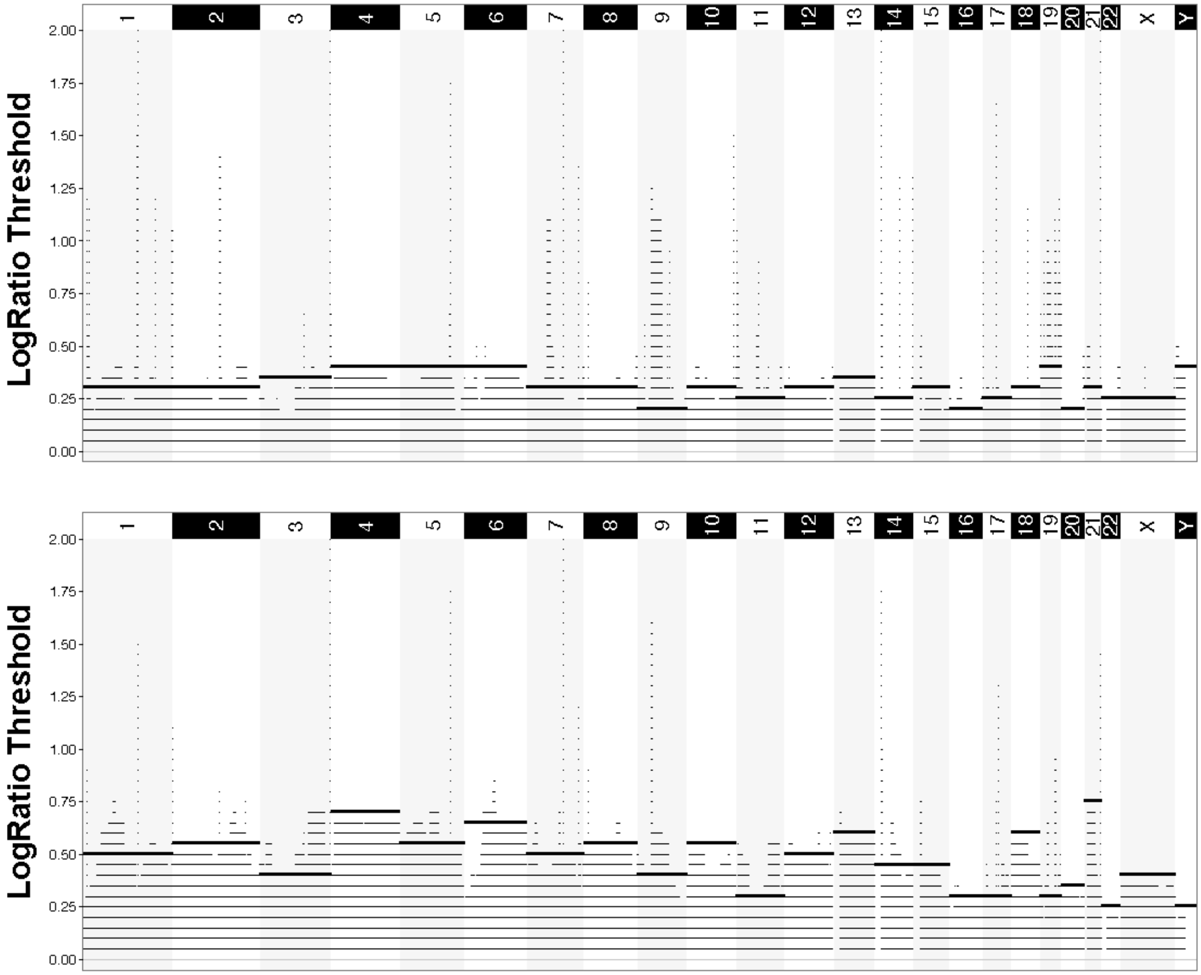

Figure S9, continued.

Dataset	Performance Estimates	Amplifications	Deletions
**AA CRC Matched Normal (N = 12)**	Median Genes Altered	3563	3490
	% Concordance with SNP Array sCNA Calls	***97.32***	***90.6***
**AA CRC Pooled Normal (N = 12)**	Median Genes Altered	5230	2845
	% Concordance with SNP Array sCNA Calls	***97.72***	***92.86***

Figure S10. Performance evaluation of ENVE in matched- versus pooled-normal analysis scenarios. Table showing the performance estimates of ENVE in detecting sCNAs, as compared to SNP-array based sCNA assessment, in both the matched-normal and pooled-normal scenarios. The median number of genes altered along with the median percent concordance with SNP-array calls are provided for both amplifications and deletions. For this evaluation, significant sCNAs as detected by the Partek Suite were used for comparison.
